# The ethics of ethnographic methods in conflict zones

**DOI:** 10.1177/0022343320971021

**Published:** 2021-02-22

**Authors:** Jana Krause

**Affiliations:** Department of Political Science, University of Oslo

**Keywords:** conflict research, ethnographic methods, fieldwork, limited immersion, research ethics, transparency

## Abstract

This article examines the ethics of using ethnographic methods in contemporary conflict zones. Ethnographic research is an embodied research practice of immersion within a field site whereby researchers use ethnographic sensibility to study how people make sense of their world. Feminist, conflict and peacebuilding scholars who research vulnerable populations and local dynamics especially value ethnographic approaches for their emphasis on contextual understanding, human agency, egalitarian research relationships and researcher empathy. While immersion leads to knowledge that can hardly be replaced by using more formal approaches, it also elicits ethical dilemmas. These arise not only from the specific research context but also from who the researcher is and how they may navigate violent and often misogynous settings. I argue that many dilemmas may and perhaps should not be overcome by researcher skill and perseverance. Instead, ethical challenges may lead researchers to adopt *limited* and/or *uneven immersion* in their field site, not as failed or flawed ethnography but as an ethical research strategy that incorporates ethnographic sensibility to a varying extent. Examining why researchers may opt for limited and uneven immersion is important because in conflict research, stereotypes of the intrepid (male) researcher with a neutral gaze still tend to mute open discussions of how gender, race, ethnicity, nationality, class and other background factors inevitably shape immersion. This article seeks to contribute to creating discursive space for these conversations, which are vital for researchers to analyse, reflect and write from the position of a ‘vulnerable observer’ and incorporate greater transparency in the discussion of research findings.

## Introduction

In political science, conflict research and peacebuilding research, recent books and articles testify to a ‘resurgence’ of ethnographic methods (Simmons & Smith, 2019; see also Autessere, 2014; Berenschot, 2011; Fujii, 2009; Mazurana, Jacobsen & Gale, 2013; Macaspac, 2018; Millar, 2018; Pachirat, 2017; Pearlman, 2015, 2017; Wedeen, 2010; Wood, 2003, 2013; among others). Ethnographic research is commonly understood as immersion in a field site, long-term engagement, and participant observation, often combined with other forms of more or less structured conversations and interviews (Bell, Caplan & Karim, 1993; Fujii, 2010; Mazurana, Jacobsen & Gale, 2013; Millar, 2018; Schatz, 2009; Wood, 2006). Researchers not only pay attention to specific questions to be answered but also immerse themselves in the broader meaning-laden context of their interlocutors (Schwedler, Simmons & Smith, forthcoming). Immersion allows for developing ethnographic sensibility beyond face-to-face encounters and interview settings. It necessitates emotional engagement to glean meanings that people attribute to their social and political reality and to understand people’s narratives in the context of their everyday lives (Yanow, 2006; Schatz, 2009). The length and depth of immersion for research to qualify as ‘ethnographic’, and the extent to which the researcher adopts an emic perspective of people’s own understanding of their actions, have remained contested. Traditionally, long research stays are the hallmark of ethnography. However, whether such deep immersion is necessarily the most ethical research approach in contemporary conflicts requires careful reflection.

This article examines the ethics of using ethnographic methods in contemporary conflicts. Feminist, conflict and peacebuilding scholars who research vulnerable populations and local dynamics of conflict and peacebuilding especially value ethnographic approaches for their emphasis on contextual understanding, human agency, egalitarian and reciprocal research relationships, and researcher empathy (Bell, Caplan, & Karim, 1993; Millar, 2018). Ethnographic peace researchers hold that analysis of, and solutions to, armed conflict must be informed by an understanding of how conflict is experienced by those who live through it (Millar, 2018). There is an extensive literature on the ethical challenges of fieldwork in conflict zones (Campbell, 2017; Cronin-Furman & Lake, 2018; Fujii, 2012; Lake, Majic & Maxwell, 2019; Mazurana, Jacobsen & Gale, 2013; Malejacq & Mukhopadhyay, 2016; Parkinson & Wood, 2015; Shesterinina, 2019; Wood, 2006) and the ethics of publishing research findings (Knott, 2019; MacLean et al., 2018; Parkinson & Wood, 2015; Schwedler, Simmons & Smith, forthcoming; Tripp, 2018). Here, I focus on the complex dilemmas and sometimes outright limitations immersive research inevitably elicits. Many dilemmas cannot be foreseen in the ethics reflection prior to departure (MacLean et al., 2018). Immersion remains an intrusion into a system of relationships long before publication (Brigden & Hallet, 2020) because the intruder is never a neutral factor but a specific person with a particular body, characteristics, personal history and political worldviews.

In some areas, ethical challenges may be so profound that research should not be conducted (Wood, 2006). In many others, however, limited and uneven immersion may be ethical and lead to crucial insights. For ethnographic research to be ethical, the duration and extent of observation necessarily needs to vary according to context. Ethical dilemmas concern the physical and emotional dangers associated with a particular field site and the researcher’s impact on the environment but also the dynamics of access and navigation as shaped by the gender, race, ethnicity, class, nationality, age and background of the individual researcher. Because ethnographic research is an embodied research practice, dilemmas arise not only from the specific conflict environment but fundamentally from who the researcher is, and is allowed to be, within this environment.

I argue that long and deep immersion may not always be the most ethical approach in many contemporary conflicts. No matter how skilled, prepared, dedicated and resilient the individual researcher is, many circumstances may or should lead researchers to opt for limited and uneven immersion. By limited immersion, I mean shorter research stays and longer breaks between exposures to the field site. By uneven immersion, I mean accepting a varying quality of access, rapport and depth of relationship with different population groups in the field (see also Hanson & Richards, 2019).

The knowledge that immersion generates – how people make meaning and exert agency – can hardly be replaced by more formal methods or fieldwork by remote control. Long and deep immersion is therefore a highly desirable research practice. However, where such research is not possible, researchers can still pursue a flexible ethnographic approach to navigate ethical dilemmas without losing some of the insights gained from this perspective. The messy realities of fieldwork, the challenges associated with violent settings, and contexts of racist and misogynous attitudes, impact researchers very differently. Rather than holding on to the traditional notion of ethnography as a masculine rite of passage and an exercise in endurance (Berry et al., 2017), a perspective that criticizes the ‘stop-and-go’ nature of limited and uneven immersion as failed or flawed ethnography, some scholars may actively choose limited and uneven immersion as an ethical research practice.

I build on scholarly arguments for integrating ethnographic sensibility into research designs even if based on shorter stays (Simmons & Smith, 2017, 2019), distinguishing various forms of ethnographic research from the classic notion of ethnography (Millar, 2018; Björkdahl & Selimovic, 2018), and on Lee Ann Fujii’s notion of ‘accidental ethnography’ (Fujii, 2015). Fujii described the term as ‘moments when the researcher is not engaged in more formal research methods, such as an interview or archival research, but in mundane tasks not specified in the research design’, which can offer valuable insights that the researchers should actively pursue (Fujii, 2015). I argue that such moments need not be ‘accidental’ but should be expected and actively pursued even during limited, relatively short, and uneven immersion.^1^ A flexible approach means that researchers need to ‘rethink tempo’ (Malejacq & Mukhopadhyay, 2016) because shorter stays in active conflict zones may not only be safer but also allow greater agency in balancing networks of informants. It further means that researchers let go of the expectation that all ethnographers become full participants in their field sites and instead value ‘uneven immersion’ as data in its own right (Hanson & Richards, 2019).

I develop this argument by building on ethnographic research from the conflict, peacebuilding, feminist and anthropological literatures. I further draw on my own fieldwork experience in central Nigeria, where I researched large-scale communal violence around the Muslim/Christian cleavage in the city of Jos and analysed the dynamics of conflict prevention within one nonviolent community (Krause, 2018). This research took place during four short research trips that together amounted to five months in the field.^2^

I first briefly lay out the ethical strengths of ethnographic research in conflict zones. I then discuss the many ethical challenges that may lead researchers to adopt limited and/or uneven immersion as an ethical research strategy in contemporary conflicts. Although researcher skill and preparedness are crucial components of effective and ethical fieldwork, I emphasize the embodied nature of ethnographic research and the gendered and racial experiences that are inevitably part of it and may (or should) not always be overcome by skill, endurance and resilience. I conclude by emphasizing the ethics of writing as a vulnerable observer to allow for a more transparent analysis of ethnographic and fieldwork-based findings.

## The ethical strengths of ethnographic research in conflict zones

Ethnography is a research method that ‘aims to study people in their own time and space’ (Koonings, Kruijt & Rodgers, 2019: 4). The knowledge gained from ethnographic sensibility and (limited) immersion – noticing, observing, and overhearing mundane aspects of everyday life – allows for grasping the complexity of social life as encountered rather than as expected, which can hardly be replaced by more formal research methods. Immersion allows researchers to develop ‘orientational knowledge’ (Elwert, 2002), both in a physical and a sociocultural sense, which is fundamental for navigating a field site ethically. Researchers learn from mundane observations in a new environment and develop a basic understanding of other people’s perspectives and the ‘ground rules’ that make up everyday life. Such knowledge develops from informal interactions and observations rather than formal interviews. It represents an important component of ethical research because it informs assessment of personal safety and safe spaces to meet respondents for more formal and structured interviews. Noticing, observing and reflecting upon one’s own emotional responses further generates knowledge and allows researchers to, at least temporarily, distance themselves from the assumptions and categories they brought to the field in the first place. Immersion and participant observation further enable researchers to study aspects of meaning-making that do not lend themselves easily to verbalization in interviews and surveys because the respondents may find them too trivial, too embarrassing, or too traumatizing to mention. The meta-data of spoken and especially unspoken expressions, such as silences, nervousness, or evasion, can signal to the attentive researcher what subjects may not (yet) be broached in a conversation (Fujii, 2015).

Ethnographic sensibility allowed me to better understand important everyday dynamics within the nonviolent community in Jos, Nigeria, and the fragility of its peace efforts in the context of ethno-religious conflict. During one of my first visits to this community, which was ethically, religiously, and socio-economically as mixed as many violence-affected neighbourhoods, I was accompanied by a local resident who worked with a peacebuilding NGO and had arranged several meetings for me that day. After several interviews, we spent some time waiting at the edge of the local market watching people’s everyday interactions. I tried to scribble notes before my next meeting when a small van stopped near us, blaring a song in the Hausa language that I did not understand. The noise was deafening and distracting, so I asked my research assistant what the music was about and what the lyrics meant. He laughed a little and then explained that it was a Christian song about how Christians would overcome Muslims and triumph, how they would prepare themselves for the fight and not be intimidated. I was shocked; this was the largest nonviolent community in Jos where Muslims and Christians had undertaken effective and extraordinary efforts to prevent killings. Yet, such offensive music was played in public, around the market place, and no one seemed to intervene. When I asked him whether the song was indeed as offensive to Muslims as it sounded to my ear (he was Catholic), he agreed that it was. He acknowledged that these things happened regularly, but people decided not to be provoked. This observation led me to conclude that nonviolent communities were hardly islands of peace and harmony, an insight that was not easy to gain from more formal interviews in which people explained the details of their prevention efforts but did not mention or tended to hide daily tensions and conflicts. Awareness of such everyday conflict dynamics allowed me to direct conversations to better understand how communities managed the often severe tensions, the burden of prevention, and the costs of becoming and remaining a nonviolent community.

When researchers try to understand local meaning-making they take people’s perceptions, sense-making and agency seriously. This allows for contextualizing and verifying information collected from more structured interviews, which can guide further interviewing. This is particularly valuable when conducting research in conflict zones, where respondents may be influenced by current political processes and by beliefs, loyalties, the impact of trauma, and expectations concerning what the researcher may want to hear. Especially for scholars with little lived experience in conflict zones – which means most foreign researchers – ethnographic insights can be vital for generating richer and more nuanced analysis of human agency. Immersion into people’s perspectives ‘slowly shifts and transforms the researcher’s own sense of what is normal and credible’ (Fujii, 2010: 240) and may also counter the often reductionist portrayals of vulnerable populations, for example women in war zones (Utas, 2005).

Ethnographic methods have some distinct advantages when weighing the ethics of research methods in conflict zones. The ethical principles developed in the Belmont Report, which guide research on human subjects, are respect for persons, beneficence and justice. Respect for persons means treating individuals as autonomous agents, requiring informed consent from research subjects. ‘Informed consent’, whether oral or written, means that research participants understand the purpose of the research, potential risks, harms and benefits to themselves and their communities before agreeing to participate (Wood, 2006). ‘Beneficence’ is understood as an obligation to making efforts to secure research subjects’ well-being. ‘Justice’ refers to the fundamental question of ‘who ought to receive the benefits of research and bear its burdens?’ (Belmont Report, 1979: 5). The principle requires the researcher’s judgement of whether the planned research entails a fair and justifiable distribution of burden versus benefit to the researched, based on reflection of power relations and vulnerability. It is intended to prevent exploitation of marginalized populations for the research benefit of the more privileged. More formal research methods may imply an unjust balance of benefit and reward for respondent and researcher because, for more vulnerable and marginalized populations, they can be burdensome and demanding of people’s time.

For vulnerable population groups, interviewing and other more formal research methods can be inappropriate and even dangerous because ‘people may be too upset, exhausted, research-fatigued, or scared to participate in a formal interview, or they may not be in a position to do so because of security, personal problems, and work, among other reasons’ (Brun, 2013: 136). For example, for a female researcher focusing on women’s wartime experience, the ethnographic ‘being there’ meant spending time with women in their family kitchens and chatting to them during everyday chores of preparing meals, which would not require the women to spare extra time (Brun, 2013: 129–148). Adopting ethnographic methods can serve the well-being of respondents better because they encounter the researcher in the context and comfort of their own daily routines. This may also provide them with a more profound sense of control over the conversation compared to the context of an interview setting, survey, or participation in a field experiment, even if the informed consent procedure for such methods is communicated with great care.

## The ethics of limited immersion in conflict zones

In conflict research, ethnography inevitably encounters limitations. The researcher would hardly join an armed group in fighting or watch a starving population as a participant observer. In many places, long-term immersion may simply be too dangerous because the security situation can change dramatically and *not* pursuing a research opportunity may be the only ethical choice (Arjona, Mampilly & Pearlman, 2018; Koonings, Kruijt & Rodgers, 2019; Schatz, 2009; Wood, 2006). Ethical conduct means respecting not only the safety and well-being of the researched but also of the researcher.

Researchers may *choose* to limit immersion for a variety of ethics-related reasons. Ethical dilemmas are difficult to generalize because they are shaped by researchers’ identities. Gender, race, age, nationality, class and other background factors, as well as personality and experience, all determine how researchers perceive their environment, conduct themselves, make judgements and are judged by respondents. In this section, I discuss how safety and well-being of the researcher, concerns over the impact of their presence, the desire to manage one’s reputation ethically, and dilemmas of intimacy, trust and betrayal may all result in choosing limited immersion.

### Dangers

Dangers, such as suspicion about who they really are and why they navigate within a conflict zone (Sluka, 2015), concern most scholars, local and foreign, but the dynamics may play out differently (Macaspac, 2018; Grimm et al., 2020). Male scholars may face physical violence, such as being beaten up by a gang (e.g. Rodgers, 2007) while female scholars have written openly about sexual assault during fieldwork (Moreno, 1995; Ross, 2015). Women researchers may tolerate risky fieldwork situations that they would not tolerate in their non-research lives (Hanson & Richards, 2017; Sharp & Kremer, 2006). Novice researchers may be particularly at risk if they worry more about methodological concerns, response rates, or doing fieldwork ‘by the book’ (Moreno, 1995; Sharp & Kremer, 2006). Male researchers may also limit immersion to keep themselves safe. A male gang researcher may decide to only conduct interviews in perilous places during the day, when gang members are not obviously engaged in criminal activities, even though not spending time with gang members at night impacts the research process and erring on the side of caution can create a frustrating distance to the lived reality of respondents (Baird, 2018: 349).

How researchers navigate violence in more mundane encounters is gendered because they are not perceived as neutral observers but as individuals with a certain social standing. Their behaviour elicits interpretation and response according to local custom. Confronting indigenous local expectations can be problematic when members of the host society may try to ‘force’ the researcher into a local gendered order. For women researchers, this can mean eliciting aggressive responses ‘in precisely the same way as local women, who wittingly or unwittingly provoke and challenge the existing order, are punished and brought into line’ (Moreno, 1995: 221). Such tensions can manifest in interactions with respondents but also with research assistants, gatekeepers, drivers and other individuals with whom the researcher regularly interacts, forms working relationships, and comes to depend upon. For male researchers, pressures or temptations to participate in ‘male bravado’ to gain access or respect within one’s field site are common in conflict settings (Theidon, 2014; Baird, 2018). However, the long-term mental health effects of such behaviour on the researcher has received little attention (Baird, 2020).

Race and ethnicity further shape the vulnerability of the researcher. While Westerners may be particularly targeted in some conflict zones, in others white researchers may experience significantly more privileged treatment than colleagues with a different racial background. A fieldwork terrain in an African country that I may experience as relatively benign while navigating it as a white woman may be unbearably saturated with sexual harassment and aggression for a female colleague with an African background. She may be confronted with local expectations of ‘proper female behaviour’ in much more direct ways than I could imagine. Thus, the emotional burden and ethical concerns researchers manage within the same environment can vary dramatically and can be deeply frustrating to convey to a wider, and often predominantly white, scholarly audience.

### Emotional impact

Researching violence is an emotional process that renders dilemmas associated with ethnography even more profound (Diphoorn, 2013). With deep listening to narratives about atrocities, loss and pain, and observations of its daily impact, the researcher often is physically and emotionally burdened (Theidon, 2014). This focus on emotional impact is not meant to disregard the suffering of those living in conflict zones who are not able to leave when their situation becomes unbearable. Discussing emotions and trauma is no unnecessary (Western) navel gazing but necessary self-care. The researcher’s trauma does nothing to alleviate the suffering of those most affected by conflict. Rather, it can do a significant disservice to respondents (Markowitz, 2019: 2).

Although long research periods continue to be seen as the hallmark when using ethnographic methods, they can undermine the research and negatively impact the researcher (Elwert, 2002). When strangers become accustomed to their field sites they can lose distance, awareness and noticing (Schütz, 1944). They may have their ability to judge dangers accurately compromised, particularly when time, financial constraints and pressure to deliver ‘good data’ encourage risky behaviour (Hanson & Richards, 2017: 9). Loneliness, feelings of anger, frustration and self-doubt do take their toll (Diphoorn, 2013; Chappuis & Jamar, 2016) and the researcher may question how ethical immersion and participant observation are when their emotional response to human suffering may rather be to abandon the project and support humanitarian aid (Wood, 2006). Fear of certain respondents, such as combatants or gang members, may also adversely affect the researcher and result in serious stress (Shesterinina, 2019). The energy needed to consistently engage with interviewees empathetically is emotionally very draining (Wood, 2006).

Long-term engagement is sometimes better achieved through multiple shorter trips. Returning to the field earns the researcher trust (Elwert, 2002; Wood, 2013; Malejacq & Mukhopadhyay, 2016). Personal and family obligations and the need for emotional support are important reasons for returning for further data gathering at a later stage. This strategy also allows for reassessing access and networks for more balance and better verification of findings (Björkdahl & Selimovic, 2018). Yet, those who are often most vulnerable to the impact of research-related trauma and burnout, doctoral students and post-doctoral researchers without secure incomes and positions, often experience the greatest pressure to remain in the field longer (Loyle & Simoni, 2017: 142).

Self-care and healthy coping strategies are vital for researchers navigating violent and misogynous settings to be neither emotionally overwhelmed nor numb. Stories of colleagues experiencing elevated levels of aggression, excessive drinking and strains in their relationships are common among scholars who conduct research in conflict settings (Loyle & Simoni, 2017). Procrastination, specifically with regard to work related to the traumatic material, is also a common response, particularly when writing leads to restimulation of the pain of people’s life histories (Theidon, 2014). Many coping strategies may remain subtle, but awareness can help the researcher turn them into healthy strategies, such as arranging adequate breaks between fieldwork and analysis of collected material. For example, I decided to only analyse difficult material for a certain number of hours per week, and only in an office setting, to keep it out of my home and limit its impact on me.

### Researcher impact

Researcher impact on local dynamics may necessitate limited immersion because their presence may affect local incentive structures, expectations and coping strategies (Goodhand, 2000: 12). For example, sites of peacemaking and peacebuilding may not always be appropriate places for immersion. When I conducted interviews in the nonviolent community in Jos and planned to spend further time ‘hanging around’ the local market observing intercommunity interaction, a resident who facilitated my meetings that day instead told me not to return for a while. Some people were uneasy about the presence of a foreign researcher because the neighbourhood was tense. Residents feared even more pressure from surrounding armed groups to give in to the conflict dynamics, join the fighting in other communities, and even facilitate an attack on part of the population within their area. BBC News had just published an article about this nonviolent community and NGO staff increasingly arrived in this neighbourhood to learn about effective prevention, thereby exposing people’s quiet violence prevention work to the whole city during a time of serious tensions. I left the neighbourhood as told, judging such conduct as ethically appropriate, despite the potential gains of more observation, because my presence could draw further unwanted attention to fragile peacebuilding efforts. I only returned more than a year later upon securing interview invitations from community leaders.

Navigating one’s own reputation ethically in the field is another reason that may require a limited presence. In public settings, the ethnographic ‘hanging around’ is a particularly visible and intrusive research method. While interviews may be held in carefully selected meeting places somewhat hidden from a larger community, the observant researcher can be seen by many. They will be assigned roles, such as being connected to aid and development workers, which can facilitate (Cronin-Furman & Lake, 2018) or prevent safe access and ethical research (Fishstein & Wilder, 2013). Professional conduct does not always prevent rumours, unwanted advances, or sexual harassment and even assault. Even if researchers introduce themselves and their work carefully, local perception is to a large extent beyond their control and may limit or foreclose immersion. Despite careful communication, a researcher’s presence may result in various rumours about ‘who she really is’ (Fujii, 2010) and cause people to fear a host of negative consequences following the ‘intruder’s’ visit (Jentzsch, 2018). The researcher may address some rumours productively and learn from them for further research (Fujii, 2010) yet others may foreclose immersion or place the researcher or the researched in danger.

Long immersion raises the risk of becoming part of the social, political and economic complexity that constitutes life in war zones in ways that the researcher may deem unethical. In some areas, increasing attacks aimed at foreigners may make venturing into certain places ever more dangerous, requiring close cooperation with specific local networks (Malejaqc & Mukhopadhyay, 2016). Yet, being seen with certain political or violent actors may convey an ethically problematic message of ‘paying tribute’ to them (Malejaqc & Mukhopadhyay, 2016), an issue that is particularly relevant for perpetrator research. I conducted my own research with perpetrators of communal violence at a career stage when I had the funds to hire a relatively expansive car with darkened windows and a driver. In Jos, Nigeria, this allowed me to meet with gang members and other former perpetrators at the edge of their neighbourhood within the car somewhat outside of people’s views. A foreign researcher meeting and ‘hanging out’ with former perpetrators in their own homes, noted and watched by neighbours, may have conveyed legitimacy or paying respect to those who have fought and killed. In this case, my generous research funds made ethical conduct possible.

How researchers understand (gendered) power structures and determine the potential benefit or harm of their presence to different population groups guides their ethical decisions for or against further immersion. There is no neutral judgement of ethical research conduct because ‘the researcher’s presence is both a boon to some and a liability to others’, which means ‘the researcher faces different ethical dilemmas with different people’ (Fujii, 2008: 19). For example, Nancy Scheper-Hughes detailed how male community leaders in her field site in South Africa told her not to return, while women from the community explicitly asked her to continue her engagement and saw it as of value and benefit to themselves (Scheper-Hughes, 1995).

### Trust and betrayal

Deep and long immersion may increase the ‘exploitative nature’ of ethnography. There is no ethically pure or purely feminist ethnography as researchers’ relationships in the field will inevitably be flawed with some degree of inauthenticity, unfulfilled expectations, potential manipulation, and emotional betrayal (Stacey, 1988). Researchers derive knowledge from relationships of trust and various degrees of intimacy. What a researcher eventually publishes may be appreciated by some but felt as betrayal by others (Fujii, 2008). A good researcher may listen, participate and observe so well that respondents forget that the researcher is primarily ‘gathering data’. He or she may also capture information that interview respondents would not voluntarily offer. Even if researchers insist that ethnographic knowledge production represents collaboration between researcher and participants, over time people may lose sight that the researcher is not only their ‘friend’, ‘trusted companion’, ‘member of their social group’, or ‘therapist’. Being increasingly perceived in a role that a researcher is unable to fulfil may warrant limited immersion. For example, in Jos, I repeatedly observed community peace meetings sponsored by various local NGOs who often had excellent grassroots connections and introduced me to community leaders. One such peace programme was conducted with high school students. I planned to simply observe from the background, but since I stood out as a foreigner, one female student demanded that I should respond to their discussion. She narrated to me in a visible state of trauma her own experience of having lost nine family members during a well-known massacre. Did I personally think that she would ever be able to forgive the perpetrators, she wanted to know. I had no good response, being unprepared for such a confrontation, and a trained member from the NGO managed the situation carefully. Thereafter, I limited my time as an observer in such programmes because ‘being there’ often meant being perceived as understanding, professional and available for social-psychological support, which I was unable to provide or promise as a follow-up.

To conclude, researchers’ safety and emotional challenges, concerns over the researcher’s impact, and potential exploitation of respondents may all lead researchers to choose limited immersion as ethical conduct. Such ethical decisionmaking may differ substantially between researchers working in similar environments because who the researcher is shapes the impact and gravity of ethical dilemmas.

## The ethics of uneven immersion

Limited immersion can generate very valuable knowledge from a position of partial embeddedness in the research context (Fujii, 2015; Malejacq & Mukhopadhyay, 2016; Schatz, 2009; Wood, 2006). *How* researchers immerse themselves, however, depends on the logistics of access, the body and background of the researcher, and on performance of gendered behaviour. Traditionally, ethnographers have viewed the dangers associated with violent contexts as obstacles to be overcome by negotiation, mediation, planning, foresight, skilful manoeuvring, impartiality and ultimately researcher adaptation (e.g. Feenan, 2002: 162; Rodgers, 2007; Sluka, 2015). All these are crucial skills and researchers necessarily adapt to the field site to some extent. However, making conscious decisions about the limits of such adaptation and uneven immersion is also part of the ethical perspective. In contemporary conflicts, such as Afghanistan or South Sudan, researchers are inevitably partial. They manoeuvre safely with painstakingly arranged affiliations to a local university, an NGO, or an international organization – all of which are political actors to various degrees – and through ‘building their tribes’, including within violent networks (Malejacq & Mukhopadhyay, 2016). Furthermore, immersion is not simply a question of skills researchers wield independently of their gender, race, ethnicity, class and other background factors. Obstacles resulting from who the researcher is may not, and perhaps should not, always be overcome by sheer persistence. Instead, ‘uneven integration’ can in itself be understood as data (Hanson & Richards, 2019: 189).

### Access

Access is shaped by the researcher’s body and performance of gendered behaviour in a specific context. While working as early career scholars in Afghanistan, Romain Malejacq and Dipali Mukhopadhyay noted how their personal characteristics determined access and expectations (Malejaqc & Mukhopadhyay, 2016: 1018). For Mukhopadhyay, the classic ethnographic ‘hanging out’, such as playing cards with commanders until late in the night, remained a male domain off limits to her, while securing more formal interviews was little trouble as long as she was ‘particularly cautious about her relationships with male counterparts’ (Malejaqc & Mukhopadhyay, 2016: 1018). In male-dominated environments, immersion can be far more difficult for female researchers because they cannot hang out inconspicuously (Brun, 2013: 136; Schwedler, 2006). Female researchers may enter private sites well and connect with more hidden population groups, such as women who do not regularly leave the house for income-generating activities, whom male researchers may find more difficult to reach.

Race and ethnicity further shape uneven immersion. Conducting ethnographic research in South Sudan as a native researcher, Jok Madut Jok found he ‘lacked the privilege of being considered ignorant and forgivable for asking absurd questions’ when ‘being able to ask stupid questions and expect brilliant answers is the stuff good anthropological research is made of, the edge an outsider researcher has over an insider’ (Jok, 2013: 157). Despite his knowledge, he had to rely on white foreigners who embodied positions of power to vouch for his credibility and support him in accessing research communities. As a male researcher focusing on sexuality, sexual violence and reproductive health, it also took him a very long time to develop trust and rapport with women (Jok, 2013: 153).

Access may further depend on the researcher *performing* gendered social practices that identify him or her as ‘one of us’, which is particularly problematic with regard to violent actors. Male colleagues, such as gang researchers, may enter the world of their informants by becoming ‘one of the guys’, quickly grab a beer, hang out in the street, an approach ‘that gives them access to spaces and conversations not open to female researchers’ (Theidon, 2014: 6). Adam Baird noted how he approached male gang members in Colombia by performing (misogynous) masculinity: ‘I would connect by leaning on my own male construction; I habitually began conversations by talking about football, beer, or women, the usual gamut of what might be called “male patter” to ingratiate myself as “one of the boys”’ (Baird, 2018: 345). However, a male researcher may rightly question at what point such adaptive behaviour becomes harmful to his mental health and whether such performance can simply be left behind when exiting the field. Furthermore, female researchers may weigh the burdens of performing ‘feminine’ behaviour in the field very differently because it may not be associated with power, status, respect, or competence and can add significantly to frustration and anxiety.

### Navigation

Intimidation is a rather common experience especially for female researchers in the field (Hanson & Richards, 2017; Moreno, 1995; Sharp & Kremer, 2006; Shesterinina, 2019). Intimidation and sexual harassment shape researchers’ engagement with their respondents. It can undermine trust and structure interaction in particular ways, leading to the avoidance of certain field sites and uneven immersion. Mundane experiences of being ‘feminized’, belittled, and having one’s knowledge and competence questioned, can complicate not only knowledge generation but also increase emotional stress. Women researchers found the greatest fieldwork challenges often lay not so much in physical dangers but in ‘gender incredibility’, meaning ‘interlocutors’ persistently assume that the statuses of ‘female’ and ‘researcher’ are incongruent or inconsistent, an experience that women reported in interactions with both men and women in the field (Huggins & Glebbeek, 2009). Being seen as a ‘woman’ rather than ‘researcher’ can obstruct access to valuable information and can result in being locally judged as ‘sexually loose’. For example, Jocelyn Viterna described keeping her distance from men except for more formal interviews in a community in El Salvador to avoid a ‘promiscuous image’ and preserve rapport with women she wanted to interview (Viterna, 2009). Maya Berry had to end long-term immersive research with gang members in El Salvador when a gang member wanted a romantic relationship as a prerequisite for further research (Berry et al., 2017). Female researchers may perform their role according to patriarchal expectations of female behaviour to gain some access but other barriers often remain and cannot simply be skilfully negotiated (Huggins & Glebbeek, 2009).

Native female researchers may face prejudice both from their home environment and in the field (Bell, 1993). Irène Bahati, a researcher from the Democratic Republic of the Congo, recounted how upon entering a community male elders exclaimed ‘Who ever heard of a woman leading a meeting when there are men present? Education has really spoiled these women. She ought to let a man speak!’ (Bahati, 2019). Such acts of feminization are difficult to overcome. Even the most skilful female researcher may find it fruitless to pursue further immersion in such a context when navigating from a position of low social status. The foreign female researcher may be able to navigate such a field site with the privilege of being foreign (and often white) but with long-term immersion may equally face the dynamics of being feminized, rendered less competent, informed, and worthy of accessing information. For both the local and the foreign female researcher in such a situation, managing considerable research funds and keeping other researchers on payroll may enhance social status and offset some of the negative gendered dynamics. However, not every researcher can conduct fieldwork with a generous budget and the challenges of gender or racial discrimination hit early career researchers with limited budgets particularly hard.

In addition, it is important to note that the performance of emotional work is undertaken ‘disproportionately by female researchers, partly because feminist methods stress the value of close and trusting relationships with research participants, and partly because traditional gender role expectation leads research participants to expect female researchers to act as confidantes and to be sympathetic’ (Bloor, Fincham & Sampson, 2008).

To conclude, a host of ethical challenges may lead researchers to accept uneven immersion as an ethical choice because not every dilemma can be overcome with skill and perseverance. It is important that researchers are able to communicate such decisions in their analysis and writing.

## The ethics of writing as a vulnerable observer

Scholars who work in active conflict zones are not neutral; they hope that their research findings have real implications for peace, civilian protection and the protection of human rights (Mazurana, Gale & Jacobsen, 2013; Longman, 2013; Parkinson, 2019), as do many of the brave individuals who invite them into their lives and invest the time and energy to explain their everyday realities. Writing about her long-term fieldwork in Peru’s conflict zones, Theidon (2014: 6) acknowledged finding the allure of fieldwork ‘undeniable’ and feeling ‘most alive’ when conducting research. Researchers’ motivations have important emotional dimensions that shape the research process as much as their emotional responses to what has been seen, heard, sensed and observed. Ethnographic research is fundamentally personal and this quality ‘cannot and should not be obscured’ (Koonings, Kruijt & Rodgers, 2019: 7).

Although such statements are frequently made, they are challenging to implement, especially for researchers not navigating with a white and male body. The traditional notion of fieldwork in violent settings as a masculine ‘rite of passage’ (Berry et al., 2017) hardly incentivizes writing research findings from the position of the ‘vulnerable observer’ (Behar, 1996). Using ethnographic methods should require researchers to routinely engage in important practices of research openness and transparency by describing how they accessed field sites, built networks and navigated challenges (Schwedler, Simmons & Smith, forthcoming). It may now be the norm in anthropology and sociology to critically discuss how gender, race, nationality, ethnicity, class and other salient characteristics affected the research process and interpretation of findings (Arjona, Mampilly & Pearlman, 2018; Diphoorn, 2013). Yet, among political scientists, this is not standard practice (Pachirat, 2017). Instead, ideas of the ‘intrepid ethnographer’ who ‘enters his site alone to fully immerse himself in his surroundings, contaminating them as little as possible’ (Hanson & Richards, 2017: 8) hold significant currency. Stereotypes of ‘lone rangers’ and ‘cowboys’ in the field (Malejaq & Mukhopadhyay, 2016) remain a widespread imaginary influencing the reception of work that draws on ethnographic methods. Such tropes also shape the scholarly reception of ethnographic work. Female scholars reported having their findings discounted based on assumptions that their interaction with violent actors would eventually hit a wall and prevent them from accessing ‘real data’ (Hanson & Richards, 2019: 162).

Open reflection of researcher positionality, and short-comings or even failures, are an important component of transparency and ethical writing (see also Arjona, Mampilly & Pearlman, 2018: 7). Scholars have advocated assessing qualitative research based on ‘the depth of the data, the coherence and consistency of the analysis, and the quality of researcher’s reflections on the position vis-à-vis participants, how she resolved ethical dilemmas she confronted, and the limitations of the research as well as its strength’ (MacLean et al., 2018). For qualitative research, transparency should mean ‘explaining the original research design and how the project unfolded’ in the field (Lake, Majic & Maxwell, 2019: 2).

However, such ethical writing may necessitate candid reflections of how the researcher’s body and background shaped the research process, which is not standard political science practice. Instead, silence about gendered and racial dynamics often remains the norm (Bloor, Fincham & Sampson, 2008). For example, the specific impact of the male body on the generation of ethnographic knowledge is rarely discussed (Theidon, 2014). Prevailing assumptions of researcher neutrality allow for only limited space for discussion of gendered research experiences and the emotional dimension of fieldwork. However, no body is ‘neutral’ (Hanson & Richards, 2017: 14). The impact of the male body and the performance of ‘male bravado’ on knowledge production are not problematized even though stereotypical masculine behaviour inevitably shapes the knowledge that is generated with research participants and may well lead male respondents to portray themselves in ways they think the male researcher expects from them. Female researchers often report issues such as sexualized interactions in the field and even anxieties over the female body ‘contaminating’ the research process (Hanson & Richards, 2017; Ross, 2015), leaving women researchers compelled to leave out gendered field experiences that may even result in a ‘disembodied’ presentation of their research. When academic publications discuss fieldwork in identity-neutral terms they undermine ethical reflections. Discursive space is vital for researchers to explain why they chose certain research pathways and networks over potential alternatives.

## Conclusions

In line with earlier work, I argued that ethnographic methods have inherent ethical strengths that are particularly valuable for research in contemporary conflict zones. The knowledge and understanding generated from ethnographic sensibility and a focus on people’s perceptions and meaning-making is not easily replaced by more formal methods. The pursuit of understanding people’s worldviews and explaining agency on respondents’ own terms reflects the first principle of the Belmont Report, respect for persons, particularly well. Further in line with earlier work (e.g. Fujii, 2015; Simmons & Smith, 2019), I argued that using ethnographic sensibility and ethnographic methods need not be limited to researchers who spend long periods of time in the field. Even though adaptation of ethnographic sensibility during shorter research stays will not replace ethnographies based on long and deep immersion, researchers should be encouraged to actively pursue ethnographic insights, also in combination with other more formal methods, such as interviews, surveys, or archival work.

Despite the inherent strengths of the methods, researchers using ethnographic methods in contemporary conflict zones do encounter significant ethical challenges and sometimes outright limitations, some of which may be less profound for more formal research methods. A researcher’s prolonged presence in certain sites of conflict and local peacebuilding may effectively harm people by drawing unwanted attention to their hidden coping, protection and survival strategies, potentially exposing them to significant risk. The dangers of a chosen conflict environment and the violent or illicit activities a specific set of respondents may engage in can necessitate limited immersion, even if it creates a frustrating distance to the lived reality of respondents. Furthermore, the emotional impact of the fieldwork can endanger the researcher and respondents if not well managed, which may lead to limited immersion as an ethical choice. The need to reassess and better balance established networks in the field site for a thorough triangulation of findings, and the ethical management of the researcher’s impact and reputation in the field, may also result in a ‘stop-and-go’ nature of fieldwork.

Field researchers necessarily adapt to their research environment. I argue that the extent and limitations of such adaptation should remain the researcher’s conscious ethical choice. These choices will be different for different researchers because ethical challenges are shaped by who the researcher is and is allowed to be within the field site. They cannot be defined independently of researcher background, race, gender, ethnicity, class and the performance of gendered behaviour. Immersion is not simply the product of a learned skillset the researcher wields independently of their embodied identity. Obstacles that result from this identity, such as gendered and racial bias, cannot and perhaps should not always be overcome by sheer persistence. Instead, researchers may choose uneven immersion to navigate a field site ethically, weighing the benefits of immersion against its challenges, such as the performance of a specific notion of masculinity or femininity for maintaining access.

From an ethics perspective, political science as a discipline needs to enhance and guard the discursive space that allows scholars to reflect about the reality of embodied research, researcher vulnerability, and the choices made in the field and their impact on the research process. Rather than being perceived as lacking rigour and transparency, the reflexivity inherent to ethnographic methods should add both to ethics discussions and transparency of research methods more generally and to assessing the validity of specific findings. Even the most resilient researcher may benefit from a more open discussion of the challenges, dilemmas, and limitations of immersion, participant observation, and ethnographic sensibility in conflict zones (see also Grimm et al., 2020). As long as researchers perceive open and candid reflections about the imperfect nature of their fieldwork as potentially detrimental to their professional reputation and the value of their findings, in part due to gendered and racist bias within the scholarly community, major obstacles to increasing research transparency will persist.

## Notes

1 I thank Diana Fu for discussion of this point.

2 The author also conducted fieldwork in a number of other contemporary conflict zones and this experience informs the article.
